# Workplace-related inhalation test – Specific inhalation challenge: S2k Guideline of the German Society for Occupational and Environmental Medicine e.V. (DGAUM), the German Society for Pneumology and Respiratory Medicine e.V. (DGP) and the German Society for Allergology and Clinical Immunology e.V. (DGAKI)

**DOI:** 10.5414/ALX02280E

**Published:** 2021-10-05

**Authors:** Alexandra M. Preisser, Dirk Koschel, Rolf Merget, Dennis Nowak, Monika Raulf, Jan Heidrich

**Affiliations:** 1 Institute for Occupational and Maritime Medicine, University Medical Center Hamburg-Eppendorf, Hamburg,; 2 Department of Internal Medicine and Pneumology, Fachkrankenhaus Coswig, Lung Center, Coswig, Division of Pneumology, Medical Department I, University Hospital Carl Gustav Carus, Dresden,; 3 Institute for Prevention and Occupational Medicine of the German Social Accident Insurance, Institute of the Ruhr University Bochum (IPA), Bochum, and; 4 Institute and Clinic for Occupational, Social and Environmental Medicine, University Hospital, LMU Munich, CPC Comprehensive Pneumology Center Munich, DZL, Deutsches Zentrum für Lungenforschung Munich, Germany; a Mandated representative of the German Society for Occupational and Environmental Medicine e. V. (DGAUM),; b Mandated representative of the German Society for Allergology and Clinical Immunology (DGAKI),; c Mandated representative of the German Society for Pneumology and Respiratory Medicine e. V. (DGP) , and; d Coordinator of the guideline

**Keywords:** occupational asthma, allergic asthma, allergic sensitization

## Abstract

Not available.

AWMF Register No. 002/026 Class S2k (*AWMF.* S2k-Leitlinie 002-026 “Arbeitsplatzbezogener Inhalationstest (AIT) – specific inhalation challenge (SIC)”. https://www.awmf.org/uploads/tx_szleitlinien/002-026l_S2k_Arbeitsplatzbezogener-Inhalationstest-AIT_2021-07.pdf [47]) 

## 1. Introduction 

This guideline describes the workplace-related inhalation test, in German named “*Arbeitsplatz-bezogener Inhalationtest (AIT)”* for the identification of allergic or immunological asthma and hypersensitivity pneumonitis (also called exogenous allergic alveolitis) in patients with symptoms such as cough, wheezing or shortness of breath in the workplace, including delayed reactions. The AIT or workplace-related inhalation test is an elaborate specific test in which the sick person is exposed in a controlled manner under laboratory conditions to an agent present in his or her workplace. Common synonyms for the workplace-related inhalation test or, in international usage, “specific inhalation challenge” (SIC) include “specific bronchial provocation testing” and “occupational-type challenge testing” [41]. 

Woitowitz (Germany 1970) [45] and Pepys (UK 1975) [24] are considered early developers of the AIT and SIC, respectively. The SIC is now well established in occupational health diagnostics; its implementation has been refined. It is an important component in the diagnosis of workplace-related asthma; it can also be used in the diagnosis of hypersensitivity pneumonitis when the diagnosis cannot be confirmed otherwise. The SIC remains the best method for identifying and documenting the allergological relevance of new working materials to the upper and lower respiratory tract. 

The concept of SIC on which the guideline is based allows the use of native agents as well as allergen solutions to detect or exclude characteristic reactions in the area of the deeper airways and the upper respiratory tract, i.e. the nasal mucosa. 

The aim and purpose of this guideline is to describe the SIC based on the current state of knowledge in occupational medicine and pneumology. It is intended to provide guidance for the diagnosis of workplace-related respiratory diseases. The SIC allows the verification of the probable causal relationship between workplace-related inhalation exposure and a respiratory or lung disease. It is therefore an important component for answering the question of whether, from a preventive point of view, it is medically justifiable for the sick person to continue to be exposed at the workplace. In addition to a review of the scientific literature, this guideline also contains practical advice on the implementation of SIC. 

## 2. Methodology of guideline development 

The guideline on SIC was compiled according to the guidelines for the development of medical guidelines of the Association of the Scientific Medical Societies in Germany (AWMF, www. awmf.org). According to the three-stage concept of the AWMF, the present guideline corresponds to the S2k evidence grading. The following professional societies were involved in the development of the guideline:

- German Society for Occupational and Environmental Medicine e. V. (DGAUM, notifying professional society)
- German Society for Pneumology and Respiratory Medicine e. V. (DGP)
- German Society for Allergology and Clinical Immunology e. V. (DGAKI)

as well as the German Respiratory League e. V. (GRL) as further professional representation. The individual mandated representatives of the professional societies are named under the authorship on the first page. 

The recommendations formulated in the course of guideline development are based on two sources. First, the assessment of existing guidelines, in particular the comprehensive recommendations on the implementation of SIC published in 2014 by a task force of the European Respiratory Society (ERS) [41] and the German guideline from 2010 [3]. Second, an updated systematic literature search conducted for the period January 2013 – June 2018 using the bibliographic search terms published by the ERS Task Force [41, Appendix A] and updated until August 2020. 

In addition to the evaluation of existing guidelines and the scientific literature, the clinical experience of the group members and theoretical considerations have also been incorporated into the recommendations. The consensus-building process took place through two face-to-face meetings of the group members on October 6, 2017 (Wiesbaden) and March 8, 2018 (Munich), repeated voting via video conferences and e-mails, and a final written circulation procedure (Delphi procedure) in accordance with the recommendations of the AWMF. 

Potential conflicts of interest were communicated to the guideline coordinators by all group members at the beginning of the guideline development and again before completion of the consensus process using the standardized guidelines of the AWMF. The information on potential conflicts of interest was reviewed and discussed in detail at the 1^st^ guideline meeting among all group members present in accordance with the AWMF rules and regulations. No conflicts of interest were identified that could influence the professional independence of the group members in the development of the SIC guideline as a whole or of individual chapters. A detailed summary of potential conflicts of interest can be found in the guideline report for this guideline [46]. The methodological procedure and the detailed process of guideline development are also presented there. 

## 3. Indications for implementation 

### 3.1 Objectives and definition of the work-related inhalation test 

The aim of the SIC is to test the specific reactivity of the respiratory tract to workplace triggers when symptoms of workplace-related asthma or hypersensitivity pneumonitis are present. This is to improve the diagnosis of workplace-related asthma and workplace-related hypersensitivity pneumonitis. 

The SIC is defined as an inhalation exposure of an individual to a substance/mixture of substances/standardized extract or a given gas concentration that is suspected to trigger or amplify an asthmatic/rhinitic or alveolitic reaction. In principle, a SIC can be carried out in the following exposure scenarios: (1) as a workplace simulation, (2) as a standardized exposure (e.g. isocyanates) or also (3) by inhalation of work substance extracts after nebulization. 

### 3.2 Indications 

**Figure 1.**
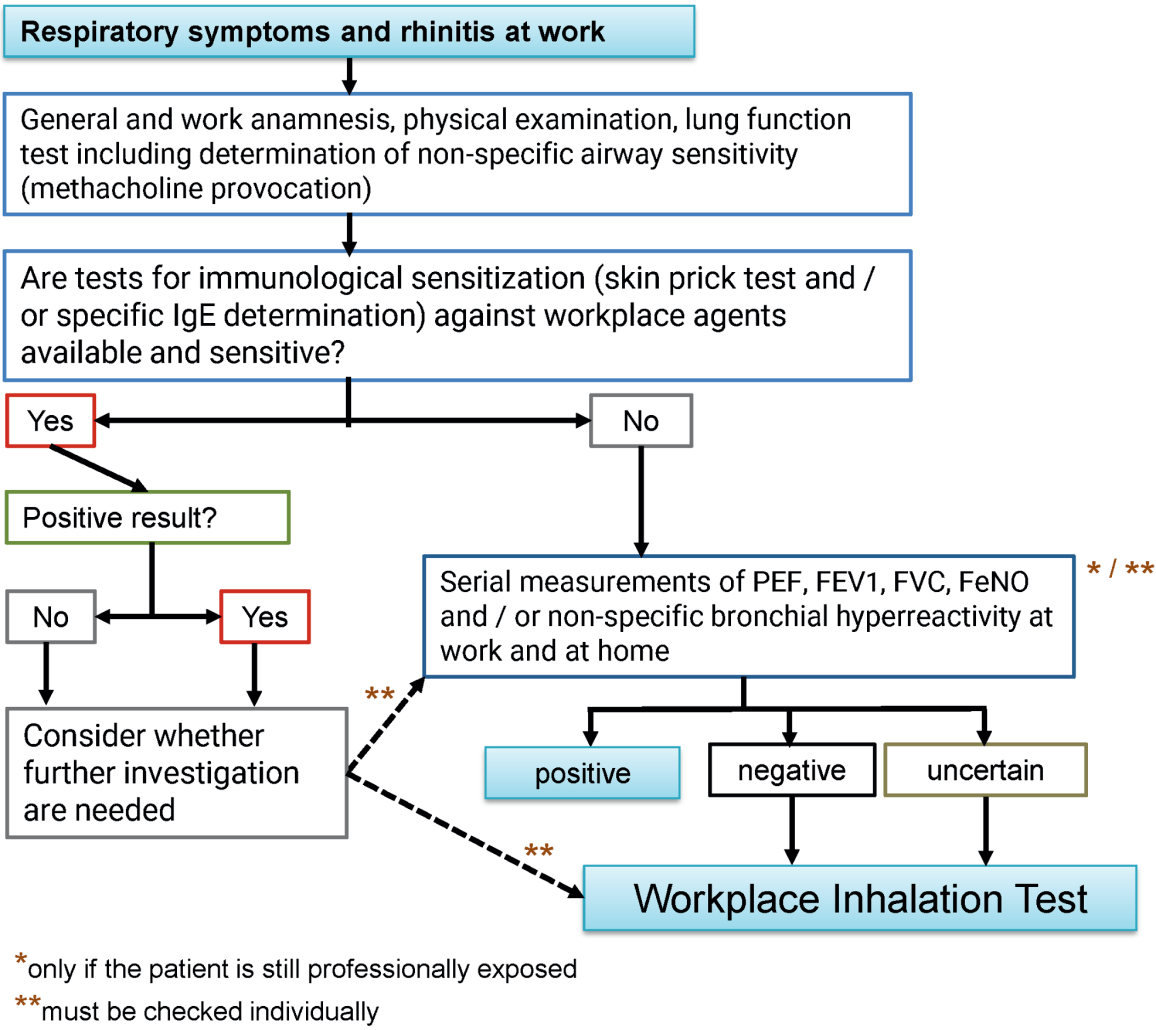
Importance of SIC in the diagnosis of occupational allergic respiratory diseases. According to [41].

The diagnosis of an allergic respiratory or pulmonary disease related to the workplace follows a stepwise approach, see Figure 1 (according to [41]). The indication is established by the assessing physician. The indication to perform the SIC is given if, in the case of clinical evidence of asthma or hypersensitivity pneumonitis with workplace relevance (work history, allergological findings, (serial) lung function diagnostics, (serial) non-specific provocation tests, workplace-related serial peak flow or spirometry measurements, if applicable, serial FeNO measurements) and if important statements for establishing the diagnosis and thus also on therapeutic or preventive measures can be expected with the SIC. This applies

- for the diagnosis of unclear workplace-related respiratory symptoms,
- if the presence of an occupational disease (Berufskrankheit, BK) is suspected, in detail:
  - obstructive allergic respiratory disease including rhinopathy (BK 4301), this also implies substances for which an immunological mechanism cannot be excluded, but which also have a chemical-irritant effect (e.g. solvent fumes, aldehydes, hairdressing substances or acrylates).
  - diseases caused by isocyanates (BK 1315)
  - hypersensitivity pneumonitis (also called exogenous allergic alveolitis) (BK 4201),
  - for the justification of measures of individual prevention according to § 3 of the German Law on Occupational Diseases (Berufskrankheitenverordnung, BKV), (here in individual cases also with the exposure to irritants)
  - as an argumentation aid for further prevention measures in the workplace.

It must always be borne in mind that the desired degree of diagnostic precision may differ for clinical, preventive medical, (accident) insurance and scientific issues, so that the examination effort and the potential examination-related risk of the patient must be weighted differently from case to case for the various issues mentioned. [Table Recommendation1]

**Recommendation 1. Recommendation1:** Recommendation 1.

SIC is indicated, - if there are clinical indications of workplace-induced asthma and/or of the presence of an occupational disease (OD) (under Nos 4301, 1315 or 4201, and in rare cases also 4302), but the diagnosis cannot be made unequivocally on the basis of other information alone, - if important statements on therapeutic or preventive measures are to be expected, including measures of individual prevention according to § 3 German BKV as well as further preventive measures at the workplace, - for the diagnosis of unclear workplace-related respiratory symptoms.

### 3.3 Contraindications 

The SIC test is contraindicated

- generally if:
  - the diagnosis can be made with simpler, lower-risk diagnostic measures with sufficient accuracy for the specific problem.
- Individually if:
  - there are symptoms of an acute inflammation or infection of the upper or lower respiratory tract,
  - a higher degree of airway obstruction is present (see Chapter 4.2),
  - (in addition) a higher-grade restrictive lung disease is present (see Chapter 4.2)
  - on the day of the examination the person has taken medication with a β-blocker or a bronchodilator,
  - extrapulmonary diseases are present that put the patient at risk, such as
    • severe cardiac diseases, especially arrhythmias
    • an uncontrolled cerebral seizure disorder
    • severe arterial hypertension,
  - a serious general illness is present,
  - there is a pregnancy,
  - the patient does not give informed consent or is unable to follow the instructions for the SIC.

## 4. Structural requirements 

### 4.1 Technical requirements 

**a. Equipment**

The following remarks refer to the performance of SIC in a designated diagnostic facility; an inhalation test directly at the workplace of the person concerned is not subject of this guideline. To carry out the test as a simulation of a workplace, it is useful to have a closed room, ideally a glazed exposure laboratory with good visibility. In this examination room, the workplace conditions are simulated with the possible allergens or triggering substances. It must be ensured that, in the event of a severe reaction, the test person can be quickly removed from this room and given further care in a room free of exposure. Preparation therefore also includes that the person puts on a disposable protective gown and a disposable hood, which can be quickly removed together with the allergens adhering to them in the event of an allergic reaction after exposure. In addition, care must be taken to minimize staff exposure through occupational safety measures and environmental contamination through unfiltered discharge of the exposure dusts or gases into the atmosphere. 

Before carrying out the test, it must be ensured that the substance suspected of being the trigger is present in the material samples with which exposure is performed. For this purpose, it is initially irrelevant whether an allergic reaction is triggered by the substance or whether the substance has an irritant effect on the bronchial mucosa due to its chemical properties, for example acrylates. One possibility is that the person concerned brings fresh samples of his or her working substances from his or her workplace or gets them himself or herself, e.g. the right type of flour, the glue actually used, the paint commonly used in the company, etc. The possible occupational substances are very diverse. The possible occupational materials are very diverse; grain flours are used most frequently, but wood dust, grain dust, flowers and plants, etc. can also be used in SIC. When using workplace materials in the tests, possible irritative effects must be avoided. When using native substances, suitable conditions with regard to sampling, containers, sealing, storage and transport should ensure that the substances can be used as fresh and unaltered as possible [12]. When testing with cereal flours or other dusts, non-allergenic tapioca flour or lactose should be used for a “negative control”. 

It must be ensured that the samples brought along by the person to be examined correspond qualitatively as well as quantitatively to the substances used at the workplace. The information required for this can be taken from the investigation reports of the accident insurance institutions, the notifications of the manufacturers or the technical safety data sheets, which must be available to the assessing physician prior to the SIC; they are part of the test protocol (DGUV, 2012). If there is any doubt about the identity of the samples brought along, the employer can be prompted via the statutory insurance provider to supply appropriate substance samples from the workplace [12]. 

Exposure to gaseous agents during SIC should be in an enclosed room with controlled supply air and exhaust ventilation, this is obligatory for isocyanates. Likewise, if practicable, the gas concentration should be measured and documented (obligatory for isocyanates). This requires the appropriate preparation of the exposure laboratory with evaporation of the gas and establishment of a controlled stable concentration of the airborne substance. Only a few centres in Germany have such exposure laboratories. 

**b. Examiner requirements, emergency drills**

The performance of a SIC requires specialist competence in the fields of pneumology and/or occupational medicine and/or allergology. In addition, the physician must be familiar with the specifics of occupational allergens, irritants and workplace-related exposure, e.g. through appropriate training at specialist conferences. SIC has the potential to trigger a severe asthma attack, and rarely also a severe general allergic reaction. Accordingly, the center carrying out the procedure must have or ensure the availability of all the appropriate emergency equipment, medications and training for staff and physicians to deal with these situations. 

**c. Patient monitoring, precautions**

The application of a venous cannula already before the start of the test, in order to be able to react directly with medication in case of a severe reaction (severe asthma attack, anaphylactic shock) should be considered individually based on the medical history and the expected reaction. Emergency instruments (for measuring oxygen saturation and blood gas analysis as well as for oxygen administration and inhalation assistance) and emergency medications for the treatment of a severe bronchial and systemic allergic reaction must be available near the examination room. These must not be used for other activities during the period of the SIC. These emergency instruments and emergency medications, as well as a nebulizer system resp. aerosol delivery device for the inhalative administration of bronchodilators must be kept in immediate vicinity. 

### 4.2 Patient prerequisites 

**a. Medical findings**

The findings known from the file brought along by the patient and collected by the physician provide information regarding the relevance and severity of proven sensitization (prick test, IgE) to noxious agents at the workplace and the already occurred or expected severity of an allergic reaction. High pre-existing specific IgE concentrations have a high predictive value for a positive test result in the SIC [43]. It is therefore necessary to check whether in these cases the indication for the SIC still exist or whether the recognition of the association and the recommendation of a BK can be made directly. The test for non-specific bronchial hyperresponsiveness is also obligatory. This result should also be considered in the indication and risk assessment of SIC. 

If there is a history of pronounced allergic reactions in previous exposures, the initial exposure dose should be very low under medical supervision. Subsequently, the exposure dose can be increased gradually. Previous findings and medical events should also be considered in the planning of SIC with regard to identifiable contraindications (see also Chapter 3.3). 

Pre-existing severe airway obstruction is essential to observe. Airway obstruction with a decrease in FEV_1_ < 70% of the predicted mean value (according to GLI (Global Lung Initiative), [26]) or an increased specific airway resistance (sRtot ≥ 1.5 kPa*s or sReff ≥ 1.3 kPa*s) (Both sRtot and sReff offer advantages in determination and interpretation, please refer to the relevant guideline [8].) represent a relative contraindication to the performance of the SIC due to the medical risk to the patient of further obstructive deterioration of lung function; therefore, the indication for SIC should be specifically addressed. The asthma should show a stable course. Some international centers consider an obstruction with an FEV1 of > 60% of the predicted mean value still sufficient to perform a SIC [41]; however, the higher risk in this case must be medically justified, e.g. if a sufficient statement cannot be achieved by nasal provocation alone. 

In addition to an obstructive ventilation disorder, a restrictive component or a gas exchange disorder may also be present, e.g. due to fibrosis or emphysema. When assessing contraindications, all the resources of the lungs must be included. Therefore, gas exchange disorder, as evidenced by a reduction in CO diffusion capacity (D_LCO_), CO transfer coefficient (D_LCO_/VA), or hypoxemia, also represent a relative contraindication for SIC. For all other contraindications, please refer to Chapter 3.3. 

**b. Medication**

Prior to SIC, respiratory medications should be suspended, if possible, to avoid skewing the bronchial response. This should be done according to their duration of action and, if necessary, in cooperation with the treating physician. This applies to bronchodilators (short- and long-acting β2 sympathomimetics, and anticholinergics), leukotriene receptor antagonists, and antihistamines. Inhaled and oral corticosteroid preparations should also ideally be discontinued at least 14 days beforehand; these can reduce a bronchial reaction and thus contribute to false negative results. 

In individual cases, if there is evidence of workplace-related symptoms or deterioration of lung function, it may be reasonable to perform SIC under (the lowest possible) inhaled corticosteroid dose which ensures:

- that the clinical picture is stable and FEV_1_ ≥ ~ 70% predicted value (or above the lower limit according to GLI predicted values)
- that any obstructive airway reaction to a working substance is suppressed as little as possible.

However, a false negative provocation cannot be completely excluded in this case, so only a positive reaction can be interpreted with certainty. [Table Recommendation2]

**Recommendation 2. Recommendation2:** Recommendation 2.

If possible, respiratory drugs should be suspended before SIC – according to their duration of action – so that their effect does not distort the bronchial response.

**c. Informed consent**

Written information about possible hazards must be explained to the patient in the medical information interview before the SIC is carried out. Written consent must be obtained from the subject/patient and a copy retained. [Table Recommendation3]

**Recommendation 3. Recommendation3:** Recommendation 3.

The following requirements are necessary to perform an SIC: - the indication for the SIC exists, - there are no contraindications, - the appropriate technical equipment and measuring instruments are kept available, - suitable and trained medical staff are available, - appropriate exposure substances are available, - after medical information has been given, the written consent of the person to be examined is available.

## 5. Carrying out the SIC/examination procedure 

### 5.1 Flowcharts of the SIC 

The following flowcharts illustrate various possible SIC examination procedures based on the recommendations of the ERS Task Force. Simplified, complex and comprehensive procedural schemes are presented. Figure 2a shows a simplified scheme (protocol A), which can be applied in case of low spontaneous variability. Figure 2b shows the default procedure (protocol B). The most elaborate variant, shown in Figure 2c, should only be applied in justified cases (protocol C). Explanations on protocol C are given in Chapter 5.3 Negative control and control day. The characteristics and indications of the different SIC protocols are summarized at a glance in Table 1. 

### 5.2 Preliminary examinations 

The extent of the required preliminary examinations is initially shown in Figures 2a, b, c and the comments in Section 4.2. The preliminary examination must take place before the SIC, preferably the day before. 

The following examination steps are required prior to performing a SIC:

- Study of the medical records and technical documentation
- Current anamnesis
- Physical exam
- Determination of the degree of sensitization (prick test and/or specific IgE determination), in case of suspected hypersensitivity pneumonitis also specific IgG antibodies. For the assessment of specific IgG antibody concentrations (if determined with ImmunoCAP), refer to the current reference values [29].
- Spirometry, body plethysmography, D_LCO,_ if hypersensitivity pneumonitis is suspected also blood gas analysis at rest and under stress, and if possible cardiopulmonary exercise testing (CPX)
- Determination of non-specific respiratory sensitivity (usually methacholine provocation)
- Measurement results on the level of exposure to the agent or qualified estimation of the inhalation exposure at the workplace
- Repeated interim medical history (especially with regard to infections, allergen exposure), auscultation and baseline pulmonary function on the day of exposure.

In addition, measurements of airway inflammation (FeNO, sputum cytology) may be considered, see Chapter 6.3. 

If the presence of hypersensitivity pneumonitis is considered as a differential diagnosis, the leukocyte count in the blood, the CO diffusion capacity and the body temperature should also be determined before, during and several times after the test; for details see also Chapter 7.9. 

### 5.3 Negative control and control day 

It is common practice to perform several pulmonary function tests (e.g. CPX, repeated methacholine test) on a preceding examination day as part of a baseline measurement, which allows estimation of spontaneous variability of pulmonary function over a day. The current ERS Task Force recommendation [41] calls for the additional day of testing with exposure to a control substance on the grounds that: (1) the stability of lung function measurements over the course of the day must be assured in order to correctly interpret changes in lung function findings after exposure (According to Vandenplas et al., 2014 [41], if the change in one-second capacity (at stable vital capacity) after exposure to the control substance was more than 10% compared to baseline, the subject should not be exposed to the agent until the asthma was stabilized by medication or exposure abstinence). (2) Further, according to Vandenplas et al. [41] it is necessary to have comparisons for any reactions to the agent to identify nonspecific irritant effects to the control substance that suggest that a reaction to the agent is also irritant. 

The Guideline Group agrees with this assessment to the extent that, in the case of unstable asthma and lung function values that fluctuate greatly over the course of the day, whether due to the severity of the asthma and/or the patient’s difficulty to cooperate, it can be of great value to know the daily course of lung function values. However, the fluctuations of the lung function values can usually also be estimated on the preceding examination day with repeated lung function examinations. However, the constellation that this variability can only be sufficiently estimated by exposure to the control substance on an additional examination day is present in only a few patients, so that this recommendation of Vandenplas et al. [41] is not generally followed; see also Figure 2a. The additional control day with exposure to control substance (Figure 2c) should only be carried out in those patients in whom

- according to the records and preliminary examinations, a large variability of lung function values over the course of a day is known or expected and/or
- an irritant effect of the agent plays a significant role in the concentration range to be used in SIC.

A further argument against a general additional examination day with exposure to a control substance is that in case of a lege artis performed SIC which is negative, i.e. does not lead to a significant obstructive ventilation disorder, a control day is not necessary and would unnecessarily tie up time and personnel resources. 

On the day the SIC is carried out, a non-specific irritation of the respiratory tract should be recorded or excluded. For this purpose, in the case of particulate substances, a negative control must be performed prior to exposure to the agent to be tested. In the case of dust exposure, “inert” dust samples are available. Commonly used examples include lactose or tapioca flour. If a pronounced reaction in the respiratory tract, that can be objectified by lung function analysis already occurs during this examination, no further differentiation is possible with the SIC and it can therefore be dropped. [Table Recommendation4]

### 5.4 Examination procedure and course of the SIC 

Measurements during and after exposure: 

The selected parameters (spirometry, body plethysmography, nasal flow if applicable, and standardized symptom score [30]) should be measured before the start of exposure and immediately (0 – 10 minutes) after each exposure dose. Pulmonary function should be measured additional after the end of exposure after 30 minutes, after every 2 hours, and between the 2^nd^ and the 6^th^ hour (observation period) ~ 1 – 2 hours. If a delayed reaction is suspected on the basis of the history or if the functional values are slowly deteriorating but have not yet reached the significance level, the follow-up measurements should be extended to 8 hours after the end of exposure, or even more in individual cases. Continuation of measurements in the evening hours can also be done by electronic spirometers; the device is explained to the patient, tested with him/her and given to him/her until the following day. 

Supplementary measurements are always taken when

- a conspicuous clinical finding is made, or
- the person examined reports an increase in respiratory symptoms.

Patient safety and practicability should be balanced here. 

Other complaints and findings, e.g. rhinitis, conjunctivitis and sneezing, should be documented, see Chapter 6. In addition, measurements of exhaled NO can be performed; an increase (usually only on the following day) can be diagnostically significant, see Chapter 6. Serial methacholine tests and determination of the eosinophil cell count in sputum have also shown abnormalities in studies and may be indicated, see Chapter 6. 

### 5.5 Working substances: Allergens, irritants 

Usually, occupational asthma triggers are sensitizing substances (leading to diseases in the sense of BK 4301) or workplace substances that have an irritant effect (leading to diseases in the sense of BK 4302), especially at higher concentrations. Respiratory sensitizing substances can be subdivided into high-molecular weight substances, mostly (glyco-)proteins of animal, plant or microbial origin with molecular weight of more than 5 kDa, and low-molecular weight substances. High molecular weight protein allergens (e.g. flours, natural latex, animal hair, mouse urine proteins, enzymes, wood dust, etc.), and only a few low molecular weight substances (e.g. platinum salts, acid anhydrides, reactive dyes) induce an IgE-mediated type I reaction. Most low molecular weight substances such as isocyanates, persulfates, aldehydes or abietic acid induce an immunological mechanism that has not yet been characterized in detail. 

The SIC aims to simulate inhalation exposure to a suspected symptom-causing agent experienced in the workplace; the nature of the working substance is decisive for the type of application in the test. In principle, the potential trigger should be applied in the form in which it is present in the workplace (e.g. as a liquid, gaseous or aerosol particle or in the corresponding chemical form (e.g. as a monomer or as a polymer). SIC can be performed – as outlined in Chapter “3. Indications” – in the following exposure scenarios: (1) workplace simulation, (2) standardized exposure (e.g. isocyanates), or also (3) inhalation of active substance extracts. 

If materials from the person’s workplace are used for testing, the available information (including safety data sheets, information from the manufacturer or information from the accident insurance institutions’ investigation reports) on the safety or relevance of the material should be observed. If mixtures are involved, an attempt can be made to use the individual components – if available – for the specific inhalation test. The advantage of this is that it is the specific working substance used at the workplace. A disadvantage is the poor qualitative and quantitative standardizability. 

If available, commercially available provocation solutions containing a sufficient amount of allergen should be used. With this procedure, a standardized exposure is ensured. On the other hand, it has to be considered that such standardized allergen solutions do not necessarily contain the allergens present in the workplace. In addition, unfortunately, fewer and fewer provocation solutions are commercially available; they are medicinal products and must therefore be approved before they can be “marketed”. This requires quality control so that, if used properly, independence of investigator and site can be guaranteed. Exposures to native substances do not allow this kind of standardization. 

If no commercial provocation solutions are available, water-soluble extracts (0.9% NaCl) can be prepared from the starting materials, especially the high molecular weight protein allergens. Prior to use, these extracts should be characterized as far as possible (protein content and protein profile, if applicable) and always sterile filtered (0.45 µm filter). The removal of endotoxins is time-consuming and usually not completely possible. However, the risk of a toxic reaction to endotoxin is low when using materials from the workplace. Tests with chemical-irritative substances may also be indicated if an immunological reaction cannot be excluded; in individual cases also under preventive medical aspects. 

For a comprehensive description of the various allergenic agents and irritants as well as the exact implementation of a SIC with these substances (exposure type, concentration and duration), please refer to the detailed overview [36]. [Table Recommendation5]

### 5.6 Exposure concentration and time intervals 

Inhalation exposures at the workplace must be simulated as accurately as possible in the SIC. When carrying out a SIC, one has to rely on the information provided by the person to be examined and the experience of the examining physicians; quantitative information on the level of occupational substance based on work area measurements can also be helpful. 

The following facts should be noted:

- When sensitizing agents or allergen extracts are used, the exposure level of the 1st stage must be selected so low that, if possible, no reaction occurs, considering medical history, workplace exposure, bronchial responsiveness and degree of sensitization (see skin prick test result and/or IgE antibody concentrations).
- A stepwise increase by a factor of two to four is recommended. This may be achieved by increasing the concentration and/or the duration of exposure.
- The time interval between the increase steps must be selected in such a way that a possible immediate reaction can be detected before the next increase. This means that a time interval of at least 10 minutes must be observed between individual concentrations of working substances.
- The highest air concentration used should be based on the occupational exposure limits of the Committee for Hazardous Substances [6] and the maximum occupational exposure limit (MAK (maximum concentration at the workplace)) of the Commission on Occupational Substances of the German Research Foundation [11]; these are usually based on an 8-hour exposure duration. This exposure duration is not reached in the context of the SIC. Therefore, higher air concentrations may be appropriate in individual cases.
- However, exceeding the “peak concentration” defined by the exceedance factor in the MAK and BAT (Biological tolerance values at the workplace) list of values with the maximum exposure time valid in this respect is only advisable in particularly justified cases.
- Air concentrations, especially of gaseous substances, should be monitored by suitable methods where possible. For isocyanates, monitoring of air concentrations is obligatory. [Table Recommendation6]

### 5.7 Exposure duration 

Exposure to the control substance should be conducted for ~ 15 min. The subsequent SIC is performed until the highest air concentration (see above) is reached or the termination criterion occurs (see Chapter 5.9). For immediate reactions, a few breaths or a few minutes may be sufficient. Usually the total exposure time to the agent is 30 – 60 minutes. Other European working groups work with exposure times of up to a maximum of 120 minutes in 1 day (in d’Alpaos et al. [2], 22% of the patients with a positive SIC reacted only after an exposure duration of 60 – 120 minutes). [Table Recommendation7]

**Recommendation 7. Recommendation7:** Recommendation 7.

The duration of exposure may be only a few breaths or a few minutes in the case of an immediate reaction; it should take a maximum of ~ 30 – 60 (– 120) minutes in total (adding up the times in the case of gradual increase).

In the recommendation of the ERS Task Force [41], it is envisaged to continue exposure on a 2^nd^ (subsequent) day if the findings on the 1^st^ exposure day are equivocal or negative and if higher doses of exposure are considered reasonable. This recommendation is probably based on the findings of D’Alpaos et al. [2]. These authors had demonstrated significant obstruction in 25% of subjects on a 2^nd^ day of exposure, i.e., after more than 120 minutes of exposure. The guideline group is critical of the conclusions of D’Alpaos et al. [2] and Vandenplas et al. [41], namely to add a 2^nd^ day of exposure (following the first). In this case, a two-day exposure to a control substance would also be required for results to be fully comparable between exposure days. However, this would reach the limits of practicability and possibly unreasonably increase the risk of false positive findings after exposure to an occupational substance, because the risk of a spontaneously (and not occupationally) deteriorating lung function from day to day increases with the number of days without medication. 

### 5.8 Nasal provocation 

Reference is made to Chapters 6.4 and 7.4. 

### 5.9 Termination criteria 

If possible, the test procedure should be carried out until the positive criterion of bronchial obstruction is reached (see Chapter 7). It has to be decided individually when to stop a test, this is especially true for so-called “near positive” reactions. Also extrapulmonary manifestations of an allergic reaction may force discontinuation. Intolerance of the test scenario on the part of the person examined must be dealt with individually; this and also the wish of the person examined in this respect can lead to termination of the test. (In the case of discontinuation at his/her request without a medical indication for discontinuation, it should be made clear to the person examined that the lack of evidence is to the claimant’s detriment). [Table Recommendation8]

**Recommendation 8. Recommendation8:** Recommendation 8.

SIC should be discontinued when the positive criteria for bronchial obstruction (see Chapter 7) are reached. Extrapulmonary reactions as well as the patient’s wish may also lead to discontinuation.

### 5.10 Outpatient vs. inpatient implementation 

SIC can be performed as part of an outpatient examination. Monitoring over several hours is required. When clarifying an obstructive airway disease, 4 – 6 hours of follow-up are usually considered sufficient (see Chapter 5.4). Late reactions are usually of only mild to moderate symptom severity and predominantly occur within 8 hours [19]). Anaphylactic reactions following inhalation exposure are very rare, for example possible with exposures to fish, crustaceans or latex [35]. The primary therapy for a grade II anaphylactic reaction with dyspnea or hypotension or grade III or IV is the administration of adrenaline [31], for example as intramuscular injection with an emergency pen. In addition, the administration of adrenaline can reduce the risk of a biphasic reaction [19]; however, there is no reliable evidence for the administration of corticosteroids. 

Continuous inpatient monitoring for at least 24 hours should be performed if hypersensitivity pneumonitis is suspected, a systemic reaction is developing and increasing, or if this development is suspected on record. Particular risks are considered to be:

- information for a highly distinctive sensitization,
- history of severe asthma attacks,
- nocturnal asthma attacks after occupational exposure.

To avoid further complications, the usual anti-inflammatory and anti-obstructive therapy should be resumed and continued after the positive criteria have been reached (see Chapter 7). Severe asthmatic reactions or expected severe late reactions (after reaching the positive criteria of bronchial obstruction) may require temporary intensification of inhalative therapy with short- and long-acting β2-sympathomimetics, possibly also anticholinergics or combination preparations with inhalative steroids. 

## 6. Measurement of bronchial response, recording of nasal symptoms and findings

The aim of a specific inhalation test with occupational substances is to confirm a bronchial reaction to exposure to a defined occupational substance in the sense of bronchial asthma. The bronchial reaction can be detected by lung functional evidence of an obstructive ventilation disorder, by the occurrence or increase of a non-specific bronchial hyperresponsiveness or by evidence of an increased inflammation of the airways. 

### 6.1 Airway obstruction (airway resistance, FEV_1_/FVC) 

Proof of obstructive pulmonary ventilation disorder is usually obtained by combined performance of spirometry and body plethysmography. The focus of the SIC is on the measurement of the one-second capacity (FEV_1_) and of the specific airway resistance (sRtot and sReff, resp.). The measurement of cooperation-dependent one-second capacity (FEV_1_) is well standardized and reproducible [22, 25]; the measurement of specific airway resistance (sRtot and sReff, resp.) is largely independent of the cooperation of the person being examined [8]. When performing spirometry, the quality criteria of the ATS/ERS (American Thoracic Society/European Respiratory Society) [22] and the German guideline on spirometry, must be observed [9]. Airway obstruction is defined here by a decrease in the FEV_1_/FVC ratio. A decrease in FEV_1_ with a simultaneous decrease in FVC (whether due to a decrease in cooperation or due to less deep breathing, e.g. reflexively in the case of irritants) is therefore not to be interpreted as a positive test. 

### 6.2 Non-specific bronchial hyperresponsiveness 

To further test the bronchial response, measurement of nonspecific bronchial hyperresponsiveness (BHR) can be performed before and, in the absence of obstructive ventilatory dysfunction, again the day after the specific agent inhalation test. The measurement is usually made using methacholine according to a 4-step protocol indicating the cumulative methacholine dose at which inhalation of the stimulant causes a drop in FEV_1_ of ≥ 20% or an increase in sRtot of ≥ 100% to at least 2.0 kPa*s [15, 21, 38]. Again, a decrease in FEV_1_ with a concomitant decrease in VC, and thus a normal FEV_1_/FVC, should not be interpreted as a positive test result. For the definition of an increase in bronchial hyperresponsiveness, please refer to Chapter 7.2. 

### 6.3 Inflammation of the airways (exhaled NO (FeNO), sputum cytology) 

Optionally, a specific inhalation test with occupational relevant substances may include measurements of airway inflammation in addition to lung function measurements of bronchial response. 

The determination of eosinophilic airway inflammation is achieved indirectly by the relatively simple and feasible measurement of the exhaled NO fraction (FeNO). An increase 24 hours after SIC of at least 13.5 ppb [13] to 17.5 ppb [18] can increase the sensitivity of SIC by detecting false-negative results, see also Chapter 7.4. 

An increase in eosinophils in induced sputum above 3% after the inhalation test may be an early marker of a bronchial response to inhalation of an occupational agent and may be helpful in identifying individuals who develop an asthmatic reaction after repeated inhalation exposure [20, 39]. 

### 6.4 Measurement of nasal breathing 

Since a nasal reaction is also considered a positive criterion in allergic obstructive airway disease (BK 4301), an objective measurement method such as anterior rhinomanometry should be used in addition to the clinical symptom score for whole-body exposures. In this regard, reference is made to recommendations of the DGAKI (German Society for Allergology and Clinical Immunology) and EAACI (European Academy of Allergy and Clinical Immunology), which are currently under revision [23, 30]. 

### 6.5 Clinical observations/symptom score 

It is recommended to document the pulmonary (shortness of breath, cough), rhinoconjunctival (eye watering, sneezing, runny nose), dermal and other systemic symptoms and findings (also of the abdomen or cardiovascular system as well as chills) of the person to be examined. 

### 6.6 Further parameters 

If hypersensitivity pneumonitis is also considered in the differential diagnosis, reference is made to the additional examinations to be performed in Chapter 7.9. [Table Recommendation9]

## 7. Evaluation and interpretation of the test results 

The aim of the SIC is to detect the development or increase of airway obstruction, bronchial hyperresponsiveness or airway inflammation. 

### 7.1 Airway obstruction 

The most common international parameter for detecting a positive response in SIC is the one-second capacity (FEV_1_) [41]. It is common practice to require a 20% decrease in one-second capacity relative to the baseline measurement [3]. In the latest ERS Task Force statement, a 15% drop in FEV_1_ is considered sufficient [41]. The guideline group generally does not follow this recommendation, but continues to consider the criterion of a drop in FEV_1_ of 20% as the relevant threshold. At a drop in FEV_1_ of 15 – 19%, an individual decision should be made whether to terminate the test. The relative one-second capacity (Tiffeneau index) should fall in parallel, the vital capacity should show largely constant values, unless a severe obstruction itself leads to a drop in vital capacity. 

Among others, symptoms such as mild dyspnea and cough should be included in the assessment. The spontaneous variability of FEV_1_ is associated with the difficulty of test interpretation, therefore it is recommended to make this criterion dependent on the spontaneous variability of lung function in the preliminary examinations or on the control day (protocol C) (see also Chapter 5.3). In any case, a measurement 10 – 20 minutes after exposure is required, since in the case of reactions of the immediate type the reaction is most severe then. 

Body plethysmography, which is particularly widespread in German-speaking countries, is particularly helpful when spirometric measurement cannot be clearly interpreted due to artefacts or in borderline cases. The positive criterion for body plethysmography is an increase in specific airway resistance (sRtot, sReff) to twice the baseline value and at the same time to at least 2 kPa*s. The body plethysmographic criterion is usually more sensitive than the spirometric one, but shows lower specificity [10, 21]. According to the Reichenhaller recommendation [12], either the spirometric or the body plethysmographic criterion can be used as an affirmation. 

All other lung function parameters are less suitable as effect parameters and should therefore not be performed (e.g. impulse oscillometry or determination of peak expiratory flow). [Table Recommendation10]

### 7.2 Non-specific bronchial hyperresponsiveness 

After inhalation with occupational allergens, nonspecific bronchial hyperresponsiveness may increase even in the absence of an asthmatic response. Two studies have shown in individual cases that persons with occupational asthma can exhibit an increase in bronchial hyperresponsiveness after SIC despite the absence of a spirometrically or body plethysmographically documented airway response [32, 38]. The gold standard here was an asthmatic response after repetitive allergen inhalation. From these data, it can be concluded that repetitive measurement of bronchial hyperresponsiveness increases the sensitivity of SIC in individual cases. A higher sensitivity in the methacholine provocation test is recommended as an affirmation of increased methacholine sensitivity, corresponding to a reduction of the cumulative methacholine dose needed to detect hyperresponsiveness by a factor of 2 – 3 [1, 7, 14, 16]. In a recently published study, the increase in bronchial hyperresponsiveness was compared with the increase in sputum eosinophils [28]. Hyperresponsiveness testing was found to be somewhat less predictive, so FeNO and sputum cytology should be included in the assessment when possible. 

### 7.3 Exhaled nitric oxide (FeNO) 

Measurement of FeNO is non-invasive, rapid and possible in almost all individuals. It is largely undisputed that the sensitivity of an increase in FeNO 24 hours after SIC is relatively low, in studies this was 0.45 for an increase of at least 17.5 ppb at 24 hours [18] and 0.52 for an increase in FeNO of 13 ppb [13]. It has been repeatedly described that increases in FeNO can occur in individuals who do not show an asthmatic response in SIC [4, 13]. The specificity of an increase in FeNO based an asthmatic response in the SIC is difficult to determine due to possible false negative findings in the SIC. By adding further parameters in the sense of an “overall rating” it could be shown that an increase of FeNO by at least 13 ppb has a high specificity for occupational asthma (of 0.9) [13]. The absolute increase of FeNO proved to be equally or even better suitable than a relative increase, so that also for reasons of practicability an absolute increase of ~ 13 or 17.5 ppb [13, 18] seems to be suitable as a criterion for significance. An increase in FeNO of ~ 15 ppb is suggested as a positive criterion in the SIC. 

### 7.4 Eosinophils in sputum 

It has also been shown for sputum eosinophils that an increase can occur after SIC without an asthmatic response [39]. Although the data are limited, the results indicate that the increase in sputum eosinophils may increase the sensitivity of SIC. One study compared the diagnostic value of an increase in eosinophilia in sputum with an increase in FeNO [17]; sputum analysis (with an increase in eosinophils in sputum of at least 2.2%) was superior to determination of FeNO (increase of at least 10 ppb), particularly in terms of sensitivity. Sputum induction and analysis are relatively complex methods and tools and thus require a not inconsiderable effort with corresponding limitations. However, in the absence of an asthmatic reaction or increase in FeNO, the additional assessment of sputum cytology or increased hyperresponsiveness in the methacholine test can indicate occupational asthma in rare cases [14]. 

### 7.5 Other bronchial inflammation parameters 

So far, there are no sufficient data for further cellular or soluble components in sputum before and after SIC. The same applies to parameters in the exhaled breath condensate (EBC), so these examinations cannot be recommended for diagnostics so far. 

### 7.6 Nasal and conjunctival reaction 

If the SIC is performed as a workplace simulation and a nasal (co-)reaction is anamnestically plausible for allergizing agents, a simultaneous measurement of a nasal reaction is recommended. A “significant positive” nasal reaction is considered to be a drop in nasal flow on one side of more than 40% at 150 Pa or a drop of more than 20% in combination with the occurrence of specific symptoms – for details of the nasal test, reference is made to the position paper of the DGAKI (ENT section) together with the DGHNOKHC (German Society for Otorhinolaryngology, Head and Neck Surgery) [30]. Symptoms such as eye burning and itching, skin itching, sneezing, coughing, dyspnea may indicate a clinically positive reaction and should be recorded with a symptom score; a score of > 2 points for “secretion”, “irritation” and “distant symptoms” (0 – 2 points each possible) is considered a positive reaction [30]. The findings of acute conjunctivitis after SIC should be documented photographically. 

### 7.7 Synoptic evaluation 

The assessment should be made individually, considering all available information. The result should be labeled as positive, questionable positive or negative. The SIC with assessment of bronchial obstruction is only one component in the diagnosis of work-related asthma and in particular allergic asthma. Even with the addition of the other parameters mentioned above, false-negative results in particular are possible. False-positive results are also possible especially in persons with highly variable airway obstruction, but are of less importance in the case of non-irritant or very low-dose substances. [Table Recommendation11]

**Recommendation 11. Recommendation11:** Recommendation 11.

The synoptic interpretation of the SIC should be done individually, considering all available information. The result should be labeled as positive, questionable positive or negative.

### 7.8 Time course of an asthmatic reaction 

The most common asthmatic reaction in the context of a SIC is an immediate reaction, which is typically clearly measurable after ~ 10 minutes and reaches its maximum after ~ 20 minutes. As a rule, the bronchial immediate reaction regresses spontaneously (almost completely or completely) within ~ 1 hour. Typical examples are reactions to high molecular weight allergens such as flours, enzymes, latex, animal epithelia, etc. [42]. Occasionally – especially after severe asthmatic immediate reactions – so-called dual reactions occur, i.e. after an immediate reaction there is initially a usually incomplete improvement, followed by a renewed deterioration of lung function, usually after 2 – 4 (up to 6) hours (so-called late reaction). Isolated late reactions, usually defined as the onset of an asthmatic reaction after more than one hour, are comparatively rare and tend to occur after inhalation of low molecular weight allergens [42]. Isolated late reactions after exposure to diisocyanates are particularly well described [33]. In contrast, acrylates typically cause exposure-congruent symptoms, initially with rhinitis, conjunctivitis and urticaria [37]. However, even with diisocyanates, a reaction beginning later than 2 hours after the end of exposure is extremely rare [33]. Not infrequently, atypical patterns occur, such as a prolonged immediate reaction (delayed regression) and a progressive reaction (progressive reaction; with increasing obstruction over time). 

### 7.9 Hypersensitivity pneumonitis 

SIC is rarely indicated in the diagnosis of hypersensitivity pneumonitis (exogenous allergic alveolitis). Evidence of hypersensitivity pneumonitis in the SIC includes both a pulmonary and a systemic reaction. In addition to the measurements already described as part of the SIC, measurement of diffusing capacity for carbon monoxide and serial capillary blood gas analysis must be performed, as well as serial determination of body temperature and leukocyte count in peripheral blood. In this regard, reference is made to the recommendations of the German Society of Pneumology [5, 34] and the EAACI [27]. 

Symptoms of hypersensitivity pneumonitis begin ~ 2 – 9 hours after the onset of antigen exposure and show their maximum expression after 6 – 24 hours. 

The following indications apply to the assumption of a pulmonary reaction:

- decrease in vital capacity by at least 20%,
- decrease in CO diffusion capacity by at least 15% or decrease in arterial partial pressure of oxygen by at least 7 mmHg,
- New onset moist rales over the lungs.

Significant systemic involvement is present when at least two of the following changes occur:

- Increase in the leukocyte count in the blood by at least 2,500/mm^3^,
- Increase in body temperature by at least 1°C,
- Chills, general feeling of illness and aching limbs.

For details, please refer to the recommendations of EAACI [27]. 

## 8. Limitations of the SIC 

### 8.1 False negative results 

False-negative results are most likely to occur if it is not possible to administer the correct quality or quantity of allergen. The problem can be minimized by investigating both factors as precisely as possible (including by the prevention services of the accident insurance institutions). Particularly in the case of complex working conditions with exposure to several noxious agents, simulation in the laboratory cannot be carried out reliably. Nor does the exposure time, which is limited to a maximum of 2 hours, fully reflect the reality at the workplace. 

A longer exposure abstinence usually does not lead to a loss of allergen reactivity, although the reactivity may decrease over time [20]. Therefore, in cases with a prolonged exposure abstinence before the SIC, the application of a higher allergen dose and additional procedures such as FeNO and methacholine challenge (potentially also sputum cytology) tests may be necessary to make the test more sensitive. 

The effect of pharmaceuticals must also be considered; therefore, before each test, it must be ensured that a relevant effect of pharmaceuticals is not (or is no longer) present (see Chapter 4.2). 

With regard to the effect of chemical-irritant substances, it should be noted that exceedances of the airborne limit values may occur at the workplace, which are responsible for the development of the present clinical picture. Under such conditions, the negative result of a SIC does not exclude a work-related causation or aggravation. In these cases, the assessment must be based on anamnesis (with local and temporal reference), other previous findings and occupational health findings. 

Exposure directly at the workplace under medical observation with accompanying serial pulmonary function measurement and/or measurement of FeNO [44] is the ideal case of an inhalation test at the workplace with real-life exposure and should be considered in any case if the insured person is still performing this activity and the anamnestic data show a work-related character of the respiratory symptoms. However, testing at work and during a non-working period is rarely feasible for practical, financial, work-related and partly legal reasons. 

### 8.2 False positive results 

When testing substances with both irritative and allergic/immunological effects (e.g. formaldehyde, cyanoacrylates, epoxy resins, colophony and others), the maximum concentration in the SIC should be selected so that significant irritation does not occur. For this purpose, it is helpful to know the toxicokinetics of the substances to be tested. 

False-positive results occur more frequently in persons with unstable asthma, therefore the spontaneous variability of the disease should be considered in the assessment and possibly preceded by a control day with testing with a control substance (protocol C), see Chapter 5.3. 

Spirometric or body plethysmographic findings are sufficient to define a positive test. If the spirometric test is positive but the body plethysmographic test is negative, the result should be viewed critically with regard to the breathing technique of the person examined. 

### 8.3 Possible complications 

The most significant adverse event associated with a SIC is a severe asthma attack. In a compilation of 335 SICs, it was shown that repeated administration of a short-acting bronchodilator was required in 12% and systemic corticosteroids were additionally used in 3% [40]. Important to avoid severe asthmatic reactions is the stepwise application with doubling, or at most quadrupling, of the concentration levels or exposure time. A sufficiently low initial dose should be selected. 

The degree of bronchial responsiveness to methacholine at baseline and a severe exacerbation at work are not associated with the risk of a severe asthmatic reaction after SIC [40]. Rarely (e.g. with fish and crustaceans or latex), anaphylactic reactions (even without asthma) occur during SIC, which is why allergen carryover should be avoided (wearing a protective suit, see Chapter 4.1). 

In all severe reactions, especially after late reactions or alveolitic reactions SIC may worsen the disease for a few days with necessary therapy for several days. SIC are not known to significantly exacerbate asthma over time. 

### 8.4 Costs 

In the statement of the ERS Task Force [41] it was stated that the costs of the SIC are probably much lower than the follow-up costs of a wrong diagnosis. Due to the complexity and possible complications of the test, it should be performed according to this guideline and without restrictions regarding a control day, the number of stages, the follow-up time and the determination of further non-invasive effect parameters, despite the relatively high costs. 

## 9. Need for research 

The working group of this guideline states, as did the ERS Task Force already in 2014 [41], that there is a need for research in the development of improved methods that improve the differentiation of bronchial reactions to irritant substances and to sensitizing agents. Approved provocation solutions for the most common occupational allergens should remain available or on the market in the future. A standardized and characterized allergen bank to be maintained by the umbrella organization of the statutory accident insurance providers would also be highly desirable. 

## 10. Summary 

Although the SIC is considered a reference method for the diagnosis of occupational asthma and work-related hypersensitivity pneumonitis, the facilities for its performance are not widely available and the test is probably currently underused. The main aim of this guideline is to harmonize workplace-related inhalation testing in Germany in line with European recommendations and harmonization efforts in Europe. The guideline contains consensus statements on the basic principles of practice and on the interpretation of the SIC, as well as practical recommendations on the minimum requirements for safe and reliable performance. It is intended to support physicians who wish to offer SIC in the diagnosis of work-related asthma and work-related hypersensitivity pneumonitis. 

## Funding 

The development of the guideline requires voluntary work by the authors. The small material and travel costs incurred were covered by the medical societies. 

## Conflict of interest 

The handling of conflict of interesst is presented in chapter 2.

**Recommendation 6. Recommendation6:** Recommendation 6.

The exposure dose (as product of substance concentration * exposure time) should be increased gradually, each with at least 10-minute breaks in between, during which the lung function measurements are performed. The highest air concentration used should be based on occupational exposure limits.

**Figure 2a.**
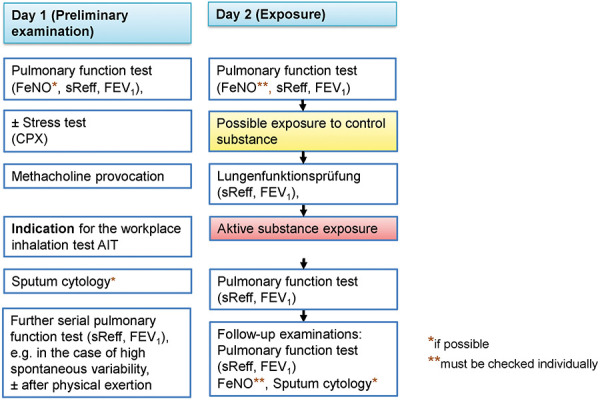
Protocol A – SIC Flowchart; simplified protocol. Indication: (1) Low spontaneous variability of lung function and (2) anamnestically or according to records justified assumption of an immediate reaction without delayed reaction to triggers at the workplace, but not to other non-specific irritant stimuli, see chapter 5.2.

**Figure 2b.**
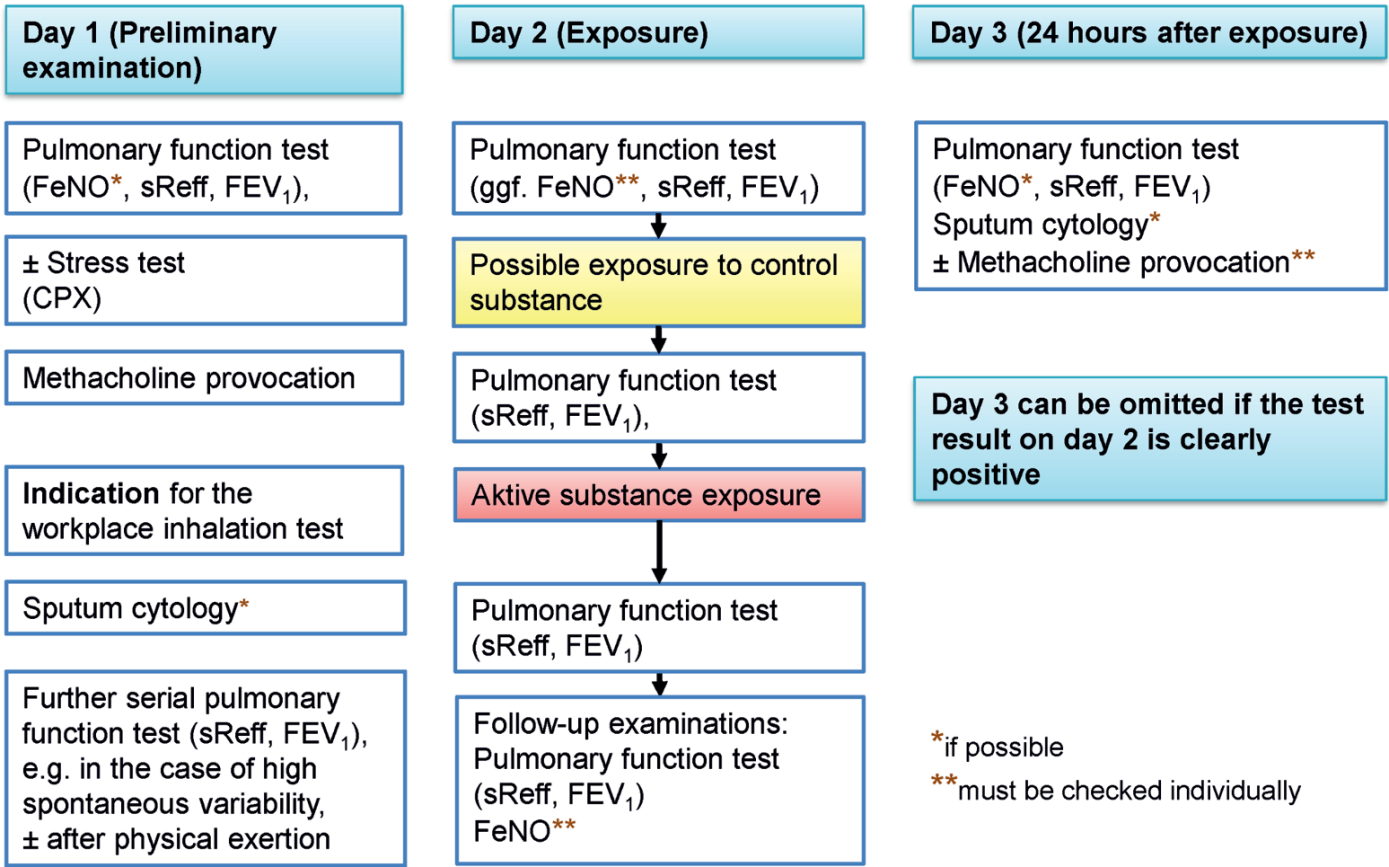
Protocol B – Standard SIC procedure; simplified protocol with late measurement. Indication: (1) low spontaneous variability of pulmonary function and (2) history and record of an acute or delayed response to workplace triggers, but not to other nonspecific irritant stimuli. Determinations of FeNO and sputum cytology should be made at least on the 1^st^ and 3^rd^ days (before and 24 hours after exposure).

**Figure 2c.**
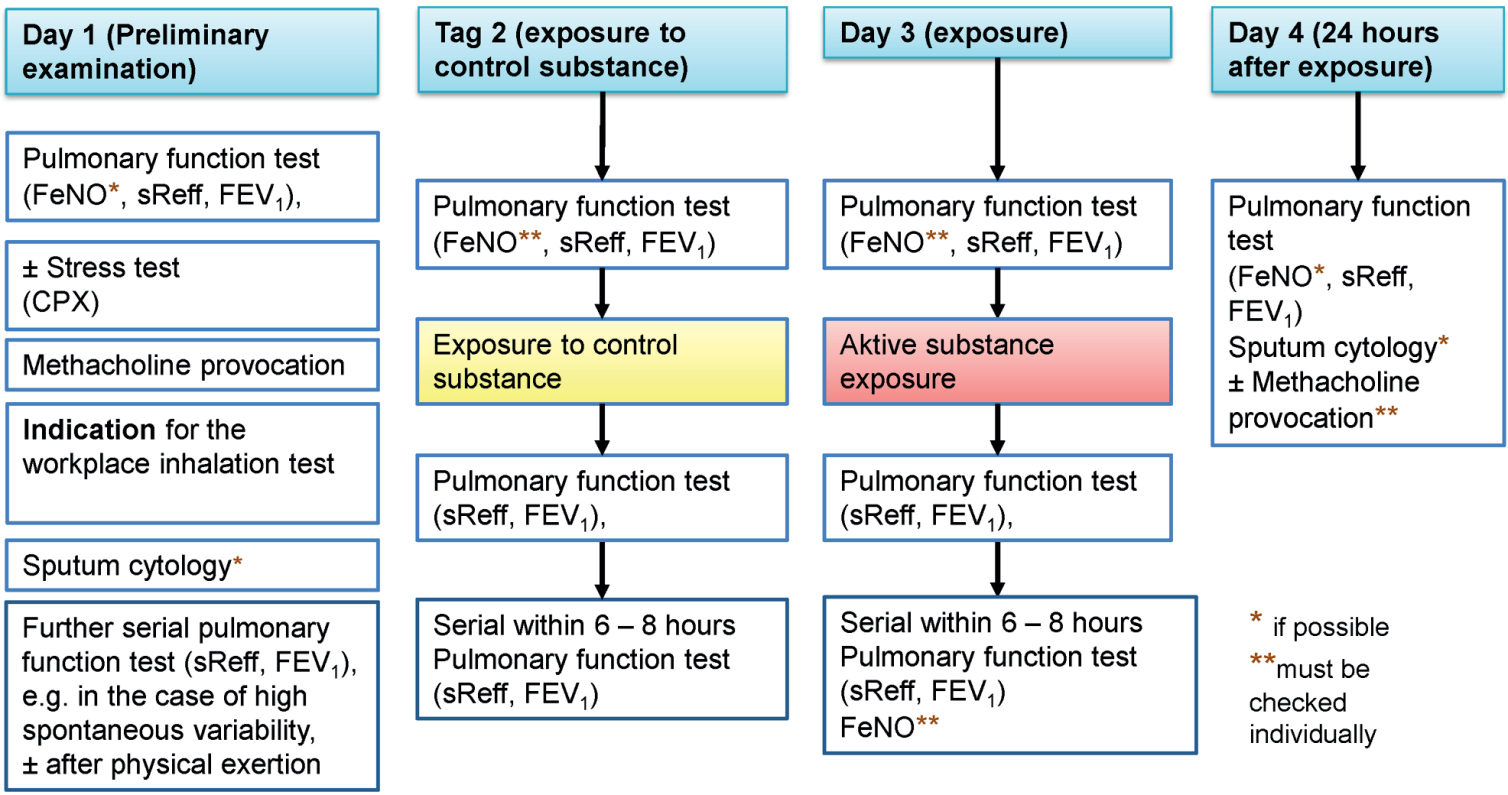
Protocol C – Flow chart of SIC in exceptional case; simplified protocol with control day. According to [41]. Indication: (1) high spontaneous variability of lung function and/or (2) anamnestically or according to records justified assumption of a non-specific reaction to irritative stimuli, without or with delayed reaction to triggers at the workplace, see Chapter 5.3. Determinations of FeNO and sputum cytology should be performed at least on the 1^st ^and 4^th^ day (before and 24 hours after exposure).

**Table 1.** SIC protocols and indications at a glance (see also Chapter 5.3).

SIC protocol	Protocol A simplified (2 days) (Figure 2a)	Protocol B with late measurement (2.5 days) (Figure 2b)	Protocol C with control day and late measurement (3.5 days) (Figure 2c)
Characteristics of the patient	Bronchial hyperresponsiveness or mild asthma	Severe asthmatic reactions, late reactions in the anamnesis	Known large variability of lung function values throughout the day
Spontaneous variability of lung function	No	No	Yes
Based on records and anamnesis, justified assumption of a non-specific reaction to irritative stimuli	No	No	Yes
Reasonable assumption of only an acute reaction on the basis of the records and medical history	Yes	No	No
Reasonable assumption, based on the file and the medical history, also of a delayed reaction	No	Yes	Possible

**Recommendation 4. Recommendation4:** Recommendation 4.

A control day preceding substance exposure is recommended especially if there is a high spontaneous variability of the lung function and/or a non-specific reaction to irritant stimuli can be assumed on the basis of anamnesis or records.

**Recommendation 9. Recommendation9:** Recommendation 9.

Essential measurement parameters of the bronchial response are the spirometrically determined FEV_1_ and the body plethysmographically measured specific airway resistance. A decrease in FEV_1_ with a simultaneous decrease in FVC is not to be interpreted as a positive result. For whole-body exposures to type I sensitizing agents, nasal flow measurements and standardized documentation of symptoms shall be supplemented.

**Recommendation 5. Recommendation5:** Recommendation 5.

For the SIC, commercially available provocation solutions should preferably be used, although these are becoming less and less available. Materials from the workplace can be used for testing; the available information (including safety data sheets, accident insurance institution investigation reports) on the safety or relevance of the material should be observed.

**Recommendation 10. Recommendation10:** Recommendation 10.

Positive criteria for SIC are present if a drop in FEV_1_ of 20% or a doubling of specific airway resistance to at least 2 kPa*s occurs with sufficient breathing technique. Care should be taken to maintain a constant value of FVC. If the test is almost positive (e.g. FEV_1_ drop of 15 – 19%), an individual decision must be made as to whether SIC should be continued.
